# *'It’s like a burden on the head'*: Redefining adequate menstrual hygiene management throughout women’s varied life stages in Odisha, India

**DOI:** 10.1371/journal.pone.0220114

**Published:** 2019-08-01

**Authors:** Elizabeth R. MacRae, Thomas Clasen, Munmun Dasmohapatra, Bethany A. Caruso

**Affiliations:** 1 Hubert Department of Global Health, Rollins School of Public Health, Emory University, Atlanta, Georgia, United States of America; 2 Department of Environmental Health, Rollins School of Public Health, Emory University, Atlanta, Georgia, United States of America; 3 London School of Hygiene and Tropical Medicine, London, United Kingdom; Università degli Studi di Perugia, ITALY

## Abstract

There has been growing recognition of menstrual hygiene management (MHM) as a significant public health issue. However, research has predominately focused on the experiences of adolescent girls in school settings. The purpose of this research is to examine detailed accounts of menstruation for women in rural Odisha, India at various life stages with a view toward improving international monitoring of MHM. Focus group discussions and in-depth interviews were conducted to understand women’s experiences of menstruation across four life stages (unmarried women, recently married women, married women, and older women). Thematic analysis was used to identify menstruation-related challenges and needs. We found women voiced needs that aligned with those captured by the WHO/UNICEF Joint Monitoring Programme for Water Supply, Sanitation and Hygiene (JMP) definition for MHM: access to clean materials, privacy for changing materials, soap and water for bathing, and disposal facilities for materials. However, we also found women require materials that are not only clean but comfortable and reliable; soap and water for more than bathing; privacy for the full spectrum of menstruation-related practices, not just when changing; and disposal facilities that are private and safe, not just accessible. Additionally, we identified needs that extend beyond the existing definition: pain management, social support, and an enabling sociocultural environment. Overall, women representing all life stages discussed menstruation challenges, including bathing, pain, and washing, drying, and storing cloth materials. Cloth management challenges were most acute for unmarried and recently married women, who were concerned that practices could reveal their menstrual status and harm their reputations, thus informing their preference for disposable materials, if attainable. We propose a revised definition of adequate MHM for this population that more comprehensively captures their needs. This definition may also prove useful for other populations, future research, creating measures of assessment, and guiding interventions and program priorities.

## Introduction

Women and girls in lower and middle-income countries (LMICs) face a multitude of barriers to safe and comfortable menstrual hygiene management (MHM). Experiencing menstruation without proper structural environments, resources, information, and support may impact women and girls’ sense of agency, self-esteem, confidence, bodily autonomy, educational experiences, and influence participation in and negative health outcomes associated with risky sexual behaviors [1–10]. Given the impacts of inadequate resources and support for MHM, addressing these needs for women and girls is a public health priority [11].

Structural challenges include inadequate provision of clean water and soap, sanitation infrastructure, private places to clean and change, and disposal facilities [7, 8, 11–22]. Resources, like commercial menstrual products, may be unavailable or too costly, prompting rural and poor women and girls to use available materials such as cloth from old clothing and cotton [11, 12, 23–26]. Improvising sanitary materials can place women and girls at risk for leaks and discomfort depending on the quantity and quality of materials accessible, and the improper cleaning and drying of these materials for reuse may potentially lead to infection, although more research is needed to substantiate this connection [27–29].

In addition to these structural and resource barriers, women and girls face information and social support challenges: girls are often unprepared, uninformed, misinformed, and lack adequate support for understanding and managing menarche, menstruation, and puberty, resulting in fear, uncertainty, and potentially harmful behaviors, like risky sex, reduced bathing, and limited food and water intake [1, 2, 7, 8, 11, 12, 21, 23, 24, 26, 30–33]. Further, the taboo and stigma around menstruation can lead to secrecy, shame, decreased mobility, social and religious restrictions, and impacts on confidence and self-efficacy [2, 11, 21, 26, 34, 35].

In recent years, the momentum to address MHM in schools has been articulated in key research, programmatic, and funding priorities [1, 11, 22, 32]. As a result, much of the literature on MHM centers on education and barriers for girls in school settings, in particular how the experience of menstruation in unsupportive environments can result in poor educational and social outcomes [11, 31, 32]. Inadequate facilities in schools for water, sanitation, private changing, and disposal, and gendered and unsupportive school environments can lead to school absenteeism and potential disruptions in academic engagement and progress [2–8, 11–13, 15–18, 21, 26, 30, 36, 37].

With the principal focus of MHM research centered on adolescent girls in school settings, considerably less attention has been given to women’s experience of MHM at other life stages, outside of a few key papers that focus on menstruation in the workplace and in emergency contexts, along with other types of vaginal bleeding [38–43]. An inclusive focus of women’s experience of menstruation—beyond adolescence in the school setting—can shed light on unique challenges, both at later life stages as girls enter new roles and in the household setting.

Studies in India, and Odisha specifically, have explored women’s MHM challenges beyond adolescence, but have focused primarily on issues related to water, sanitation, and hygiene (WASH) conditions, including sanitation insecurity, experiences with sanitation-related psychosocial stress, and reproductive tract infections [19, 28, 44–46]. Yet, menstruation concerns extend beyond adolescence and WASH. Thus, focus on women’s menstrual concerns throughout various life stages that is inclusive of, but not exclusive to, WASH concerns warrants focused attention [45, 46].

In 2012, the WHO/UNICEF Joint Monitoring Programme for Water Supply, Sanitation and Hygiene (JMP) created a definition for adequate MHM as part of their lobbying effort to have MHM in schools and health facilities incorporated into the goals, targets, and indicators of the post-2015 Sustainable Development Goals (SDGs): “Women and adolescent girls using a clean menstrual management material to absorb or collect menstrual blood, that can be changed in privacy as often as necessary for the duration of a menstrual period, using soap and water for washing the body as required, and having access to facilities to dispose of used menstrual management materials. They understand the basic facts linked to the menstrual cycle and how to manage it with dignity and without discomfort or fear” [47]. As noted by Sommer et al (2015), this definition is important as it “provides a center around which efforts can coalesce” [11]. As such, it is imperative that the definition is widely applicable such that coalesced efforts are effectively targeted and evaluated. Given that this definition was created largely in the effort to include MHM in the SDGs specifically for WASH in schools and health care facilities, exploring the applicability and comprehensiveness of the definition for women in household settings is warranted. This research aims to (1) contribute a deeper and broader understanding of women’s menstrual hygiene experiences, practices, and concerns at different life stages in rural Odisha, India, (2) understand if and how women’s MHM needs in this population may extend beyond the JMP definition, and (3) propose a conceptual framework and revised definition for adequate MHM for this population. This exploration of women’s needs and experiences of MHM, and how they inform understandings of adequate MHM, can expand the discourse surrounding MHM in LMICs and has the potential to inform future research and programming, both locally and globally.

## Methods

### Data source

These data were collected in rural Odisha, India in March-April 2014 as a part of a broader qualitative study to understand women’s overall concerns related to sanitation and associated behaviors, including urination, defecation, and menstruation at various life stages, and influences on mental health outcomes [19, 46, 48]. This specific paper focuses attention on menstruation, highlighting the breadth of women’s experiences, practices, and concerns—including those beyond WASH—and noting how they may vary at different life stages.

### Setting

Odisha has a population of 42 million people and is one of the poorest districts in India (33% of the population live in poverty) [49, 50]. Ninety percent of households do not have piped water and 65% of the population does not use a sanitation facility [51]. The state has high gender disparities, including less access to economic opportunities for women compared to other low-income states, and in most households, males hold the power for sanitation-related decisions [49–52]. For women aged 15–24, 47% use some method of “hygienic menstrual protection” (e.g., locally prepared napkins, sanitary napkins, and tampons) [51]. Of those, 69% use cloth as menstrual protection, 34% use sanitary napkins, 12% use locally prepared napkins, and 2% use tampons [51]. The use of hygienic menstrual protection methods increases with levels of schooling and 41% of Christian women, 47% of Hindu women, and 63% of Muslim women use “hygienic menstrual protection” [51]. According to a systematic review, only half of girls in India reported being informed about menstruation prior to menarche and a quarter of girls reported missing school during menstruation [8].

### Participants

Participants come from 12 communities, which were previously involved in a cluster randomized trial evaluating the impact of a rural sanitation intervention (clinical trial registration no. NCT01214785) [53]. Communities were purposively selected for variation by previous intervention status, toilet coverage, water access, and geographic conditions.

Participants were eligible for participation if they were over age 18. Women were purposively sampled for variation in life stage: (1) unmarried women (UMW) living with their parents, (2) women who had recently (past 3 years) married (RMW), (3) women married (MW) over 3 years, and (4) women older than 49 years (OW). Sampling across these categories was believed to capture diversity of MHM experience. Participant recruitment was facilitated through contacts in the communities. Specifically, for FLIs, Research Assistants and would knock on doors to determine if women were eligible and available. They would ask community members to help identify women who fit the specific categories, particularly unmarried women over age 18 and recently married women, who were the most challenging to find. For FGDs, Research Assistants called contacts in the communities before the visit, typically an Anganwadi (early child care center) worker to gather women to participate.

### Data collection and preparation

Data were collected through free-list interviews (FLIs) and focus group discussions (FGDs) by two female Research Assistants in their late twenties from Odisha who were fluent in English and Odia, the local language. One was experienced in qualitative methods while the other was an experienced journalist. Both were engaged in a multi-day training on qualitative methods and research ethics.

FLIs were conducted in eight communities and FGDs were conducted in four different communities (12 total) to enable new voices and opinions to be represented in the data. Demographic data about each participant and information about their menstruation practices were collected through a brief survey of each participant prior to the start of each FLI and FGD.

#### Free-list interviews

Interviews utilized a free-listing activity to elicit MHM concerns and understand how those concerns were experienced among a group of participants. Specifically, women first were asked to list the concerns that “women in this community” experienced while menstruating, and they were encouraged to explain each of the concerns noted. The Research Assistants then probed to uncover further variation, asking specifically if there were specific concerns related to menstruation at night and menstruation during the monsoon season. The tool also included questions about women’s general concerns and concerns related to water, urination, defecation, and keeping clean, with similar probes about nighttime and seasonality. Some of these data are reported elsewhere [19]. Throughout the interviews, the women provided detailed accounts of their MHM practices, including stories and examples. The data for this sub-study emerges from these in-depth descriptions. A minimum of 64 one-on-one interviews, with two women of each life stage per community, was targeted due to the recommendation of 30 interviews for free-listing data collection as well as the variability in the sample [54]. Interviews were conducted in private spaces over a duration of 30 to 90 minutes, though covered topics unrelated to menstruation as well.

#### Focus group discussions

FGD tools were developed subsequent to the findings from the free-list interviews. The FGDs were conducted to gather greater detail about women’s concerns and determine if the concerns were normative within the community. Like the FLIs, women were asked to list concerns women in the community had related to menstruation, and Research Assistants then probed regarding concerns specific to menstruation at night or during the monsoon season. Unlike the FLIs, the FGD had a checklist that contained the most frequently noted concerns from the FLIs. Research Assistants would check off if a concern previously identified was brought up by participants in the FGD. They would also write in if a new concern was noted that was not on the list. Once concerns were listed, the Research Assistant asked the participants to provide more details about the concerns they listed one by one. The Research Assistant then probed on concerns identified in the FLIs that the group did not organically mention on their own, asking if these too were concerns and if they could provide details and insights.

A total of eight FGDs were held throughout four communities: four for UMW and four for women who were ever-married (RMW, MW, OW), with 5–7 participants each to allow them to comfortably share on the sensitive topic [55]. FGDs lasted for one to two hours, inclusive of menstruation and non-menstruation related topics. FGDs took place in private spaces, such as schools, temples, or houses.

### Data management and analysis

Interviews and FGDs were recorded digitally and directly translated and transcribed into English. Memos were added to the data in MAXQDA to organize the analytic process, develop codes and emerging themes, and capture nuances in the data. A codebook was created using deductive and inductive codes and refined throughout the coding process; codes were applied to the transcripts in MAXQDA. Coded text was retrieved topically and explored by the subgroup of life stage (UMW, RMW, MW, OW). We used thematic analysis to categorize codes and identify themes that emerged from women’s lived experiences and challenges with menstruation. The resulting themes and issues are described and presented utilizing the JMP framework. A revised definition for adequate MHM and a conceptual framework, based on this sample, are proposed [47].

### Ethics

The Emory University Institutional Review Board (Atlanta, Georgia, USA; IRB00072840) and KIIT University Ethics Review Committee (Bhubaneswar, India; KIMS/KIIT/IEC/795/2014) approved study protocols. KIIT University reviewed and provided local approval of all protocols, with consideration to both ethics and local rules and regulations. Women provided oral consent prior to participation. Oral consent was approved as it was anticipated that not all women in the communities engaged would be literate and asking them to sign a document they are not able to read could instigate feelings of vulnerability. Verbal consent was digitally recorded after the participant provided permission to turn on the recorder.

## Results

### Participant characteristics

Data from 68 of 69 FLIs (16 UMW, 12 RMW, 22 MW, 18 OW) and 8 FGDs (each group had 5 to 7 participants; 23 UMW, 16 MW, 7 OW) were included in the analysis. One interview with an OW was excluded because she did not complete the portion of the interview related to menstruation because she needed to attend to a domestic matter. In the FLIs, women’s ages ranged from 18 to 75, 100% were Hindu, 76% had at least some primary education, 63% had water within their household compound, and 54% had a toilet within their household compound (Table 1). FGD participants were 18 to 70 years old, 98% were Hindu, 98% had at least some primary education, 70% had water within their household compound, and 59% had a toilet within their household compound (Table 1). Recently married women did not participate in the FGDs, as their family members did not give permission.

**Table 1:** Demographic information for participants in free-list interviews (N = 68) and focus group discussions (N = 46).

		All		1. Unmarried (UMW)		2. Recently Married (RMW)		3. Married (MW)		4. Over 49 (OW)	
	**Free-List Interview Participants**	68		16	24%	12	18%	22	32%	18	26%
Intervention Community (vs. Control)		27	40%	5	31%	4	33%	9	41%	9	50%
Age^1^		36.1	(18–75)	20.8	(18–27)	23.2	(20–27)	34.0	(24–47)	60.8	(50–75)
Education											
	*None*	16	24%	0	0%	0	0%	4	18%	12	67%
	*Some Primary*	17	25%	1	6%	3	25%	7	32%	6	33%
	*Some Secondary*	28	41%	10	63%	9	75%	9	41%	0	0%
	*Some Tertiary*	7	10%	5	31%	0	0%	2	9%	0	0%
Below Poverty Line Card^2^		55	85%	14	88%	11	100%	15	75%	15	83%
Hindu		68	100%	16	100%	12	100%	22	100%	18	100%
Caste^3^											
	*Brahmin*	4	6%	1	7%	0	0%	2	9%	1	6%
	*General Caste*	44	67%	12	80%	8	73%	12	55%	12	67%
	*Scheduled Caste (SC)*	5	8%	0	0%	0	0%	3	14%	2	11%
	*Other Backward Caste (OBC)*^*4*^	11	17%	2	13%	3	27%	4	18%	2	11%
	*Scheduled Tribe*	2	3%	0	0%	0	0%	1	5%	1	6%
Water Source Within Compound		43	63%	12	75%	7	58%	13	59%	11	61%
Toilet Within Compound		37	54%	10	63%	9	75%	9	41%	9	50%
	**Focus Group Discussion Participants**	46		23	50%			16	35%	7	15%
Intervention Community (vs. Control)		22	48%	10	43%			7	44%	5	71%
Age^1^		30.8	(18–70)	19.2	(18–23)			34.8	(20–45)	59.7	(51–70)
Education											
	*None*	1	2%	0	0%			0	0%	1	14%
	*Some Primary*	13	28%	0	0%			8	50%	5	72%
	*Some Secondary*	12	26%	5	22%			6	38%	1	14%
	*Some Tertiary*	20	44%	18	78%			2	12%	0	0%
Below Poverty Line Card^2^		29	67%	16	70%			10	71%	3	50%
Hindu		45	98%	22	96%			16	100%	7	100%
Caste											
	*Brahmin*	1	2%	1	4%			0	0%	0	0%
	*General Caste*	30	65%	12	52%			11	69%	7	100%
	*Scheduled Caste (SC)*	8	17%	5	22%			3	19%	0	0%
	*Other Backward Caste (OBC)*	7	15%	5	22%			2	13%	0	0%
	*Scheduled Tribe*	0	0%	0	0%			0	0%	0	0%
Water Source Within Compound		32	70%	16	70%			11	69%	5	71%
Toilet Within Compound		27	59%	14	61%			8	50%	5	71%

^1^ Not all women knew their age, some guessed. Bracketed numbers represent the full range of ages.

^2^ Missing data for 3 FLI women; Missing data for 3 FGD women.

^3^ Missing data for 2 FLI women.

^4^ “Other Backward Caste (OBC)” is the terminology of the government of India (for additional information, see here: National Portal of India. (2018). Central list of Other Backward Classes by Ministry of Social Justice and Empowerment. https://www.india.gov.in/central-list-other-backward-classes-national-commission-backward-classes-0). We elected to present the castes using this terminology, as they would be most consistent with terms used by the Government of India.

Of the 68 interviews, 66 indicated concerns related to menstruation (one MW and one OW reported no concerns related to menstruation). Sixty-nine percent of FLI participants and 80% of FGD participants were still experiencing monthly menstruation at the time of the study (Table 2), this included all participants who were unmarried and recently married, and the majority of women married over three years (86% in FLIs, 88% in FGDs). However none of the older women (over age 49) were still experiencing menstruation, but still provided insights about their experiences when they were menstruating.

**Table 2:** Menstruation information for participants in free-list interviews (N = 68) and focus group discussions (N = 46) collected from the demographic survey.

		FLI Participants										FGD Participants							
		All		1. Unmarried (UMW) (n = 16		2. Recently Married (RMW) (n = 12)		3. Married (MW) (n = 22)		4. Over 49 (OW) (n = 18)		All		1. Unmarried (UMW) (n = 23)		3. Married (MW) (n = 16)		4. Over 49 (OW) (n = 7)	
Experiencing Monthly Menstruation		47	69%	16	100%	12	100%	19	86%	0	0%	37	80%	23	100%	14	88%	0	0%
Materials Used for Menstruation^1^																			
	*Cloth*	37	54%	4	25%	1	8%	15	68%	17	94%	23	51%	3	13%	13	87%	7	100%
	*Pad*	9	13%	2	13%	3	25%	3	14%	1	6%	8	18%	8	35%	0	0%	0	0%
	*Both Cloth and Pad*	22	32%	10	63%	8	67%	4	18%	0	0%	14	31%	12	52%	2	13%	0	0%
	*Other*	0	0%	0	0%	0	0%	0	0%	0	0%	0	0%	0	0%	0	0%	0	0%
Latrine Used During Menstruation^2^		19	66%	6	60%	8	89%	5	63%	0	0%	21	100%	14	100%	7	100%	0	0%
Latrine Use During Menstruation for Urination^3^																			
	*Always*	1	6%	0	0%	1	13%	0	0%	0	0%	5	24%	3	21%	2	29%	0	0%
	*Sometimes*	0	0%	0	0%	0	0%	0	0%	0	0%	3	14%	3	21%	0	0%	0	0%
	*Never*	15	94%	4	100%	7	88%	4	100%	0	0%	13	62%	8	57%	5	71%	0	0%
Latrine Use During Menstruation for Defecation^3^																			
	*Always*	12	75%	2	50%	8	100%	2	50%	0	0%	18	86%	12	86%	6	86%	0	0%
	*Sometimes*	0	0%	0	0%	0	0%	0	0%	0	0%	3	14%	2	14%	1	14%	0	0%
	*Never*	4	25%	2	50%	0	0%	2	50%	0	0%	0	0%	0	0%	0	0%	0	0%
Latrine Use During Menstruation for Bathing^3^																			
	*Always*	0	0%	0	0%	0	0%	0	0%	0	0%	0	0%	0	0%	0	0%	0	0%
	*Sometimes*	1	6%	1	25%	0	0%	0	0%	0	0%	1	5%	1	7%	0	0%	0	0%
	*Never*	15	94%	3	75%	8	100%	4	100%	0	0%	20	95%	13	93%	7	100%	0	0%
Latrine Use During Menstruation for Cleaning Cloth/Pad^4^																			
	*Always*	2	14%	0	0%	1	14%	1	25%	0	0%	1	5%	1	8%	0	0%	0	0%
	*Sometimes*	1	7%	1	33%	0	0%	0	0%	0	0%	6	32%	4	33%	2	29%	0	0%
	*Never*	11	79%	2	67%	6	86%	3	75%	0	0%	12	63%	7	58%	5	71%	0	0%
Latrine Use During Menstruation for Changing Cloth/Pad^5^																			
	*Always*	3	20%	0	0%	2	29%	1	25%	0	0%	2	10%	2	14%	0	0%	0	0%
	*Sometimes*	0	0%	0	0%	0	0%	0	0%	0	0%	4	19%	4	29%	0	0%	0	0%
	*Never*	12	80%	4	100%	5	71%	3	75%	0	0%	15	71%	8	57%	7	100%	0	0%

^1^ Missing data for 1 FGD woman.

^2^ 39 FLI and 25 FGD women excluded from Table 2 because they had no latrine or were no longer menstruating.

^3^ Missing data for 20 FLI women and not applicable to 32; Not applicable to 25 FGD women.

^4^ Missing data for 20 FLI women and not applicable to 33; Not applicable to 27 FGD women.

^5^ Missing data for 20 FLI women and not applicable to 34; Not applicable to 25 FGD women.

### Menstruation experiences by life stage

There were variations and similarities in women’s practices during menstruation by life stage categories. Specifically, women’s material use varied by life stage, but the manner in which they used their latrines during menstruation was fairly uniform. This section reports on key differences identified across the life stages, though differences are also noted in the proceeding sections that engage the JMP definition.

Overall, according to the pre-qualitative survey, 54% of women in FLIs and 54% of women in FGDs indicated that they used cloth for managing menstruation, though these percentages were primarily driven by large proportions of married (68% in FLIs, 87% in FGDs) and older (94% in FLIs, 100% in FGDs) women who reported using cloth exclusively (see Table 2). A mix of cloth and pad use was the most common practice reported by unmarried (63% in FLIs, 52% in FGDs) and recently married (67% in FLIs) women. Exclusive use of disposable pads was reported by only a minority of participants (13% in FLIs, 18% in FGDs), most often by unmarried (13% in FLIs, 35% in FGDs) and recently married (25% in FLIs) women. During the FLIs and FGDs, unmarried and recently married women explained a specific preference for pads, whether they were able to use them exclusively or not. Ease of pad use and challenges with washing, drying, and storing cloth materials were common explanations for their preferences.

Among the women that had latrines and were still menstruating, 66% of FLI participants and 100% of FGD participants claimed to use their latrines during menstruation, but further questioning revealed that latrine use and non-use varied by activity. Most women reported always using their latrine during menstruation for defecation (75% in FLIs, 86% in FGDs). However, among FLI respondents, 94% indicated they never used their latrines for urination while menstruating compared to 62% of FGD respondents. Latrine use for urination was reported to always be practiced by unmarried (21%) and married (29%) women in FGDs. Despite the potential privacy that a latrine could afford them, almost all women reported never using the latrines for bathing (94% in FLIs, 95% in FGDs), and most reported never using it for cleaning (79% in FLIs, 63% in FGDs), or changing materials (80% in FLIs, 71% in FGDs).

Differences between life stage also emerged through the qualitative components of the study. Concerns about access to materials were primarily expressed by UMW and RWM as they, more than MW and OW, preferred pads. Much of this preference stemmed from challenges related to cleaning, drying, and storing reusable cloth.

Challenges associated with the disposal of menstrual management materials were felt acutely by RWM, many of whom had to adjust to new living environments and added restrictions in mobility due to their new marital status. RMW shared, more than women in other life stages, concerns about finding locations to dispose of and hide materials, when no formal disposal location was available. UMW and RMW also reflected on concerns about finding private locations and times to change materials.

However, many challenges related to menstruation were shared by women across the four life stages. All groups expressed concerns about washing, drying, and storing cloth used for menstruation, along with managing bathing practices during menstruation. Additionally, women in all life stages shared detailed accounts of menstrual pain and discomfort. All of these challenges were shared by OW who were no longer menstruating, indicating these issues were persistent across life stage.

### Requirements for menstrual hygiene management according to the JMP

The qualitative results are presented using the JMP definition for MHM as a framework, specifically documenting women’s concerns and experiences with clean materials, privacy for changing, soap and water for bathing, and disposal [47]. We then explore additional concerns, needs, and experiences with menstruation that emerged from the data and extend beyond the JMP definition.

**“Women and adolescent girls using a clean menstrual management material to absorb or collect menstrual blood.”** According to the JMP, one requirement for MHM is the use of clean menstrual management materials and we found this was a felt need among participants. UMW and RMW expressed preference for pads (referred to as “sanitary napkins” by participants) more than other women. OW reflected on the preference shift for pads among girls and shared how they used cloth for menstruation. Generally, women felt cloth was problematic to clean, dry, and store, and wanted to use pads instead, which could be disposed of after use. Women also worried cloth would become dirty between periods and came up with practices for storing and assuring cloth cleanliness before use:

*They have to be washed and then we fold it and keep it in polythene*, *so that no insects can enter…Or we put in the straw roof*. *And again when “mens” [menstruation] happens*, *we shake it a little for the fear that insects may have attacked…We shake it*, *wring it and after spiting 2–3 times*, *we wear it…It has to be washed and kept…So again*, *while wearing*, *we spit in it*, *shake it and see if it is clean or not and then wear*. *(FLI*, *MW*, *Toilet)*

**“That can be changed in privacy as often as necessary for the duration of a menstrual period.”** Participants reported the need for privacy to change materials. However, among women who had access to latrines, the majority (80% for FLI participants and 71% of FGD participants) reported never using this space for changing cloth or pads despite the privacy that may be afforded inside (Table 2). Predominately, women changed materials inside the house. UMW faced difficulties changing materials due to crowded homes with limited private spaces of their own. Women would actively search for a vacant place to change; one OW recalls changing in the cow shed in the backyard when she was younger. After defecating in the open, one UMW reported hiding her used cloth in her underwear to walk home and change.

**“Using soap and water for washing the body as required.”** Women in this population noted challenges related to the use of soap and water for washing the body. A few women mentioned challenges accessing soap: one MW reflected on the absence of soap during menstruation and three other women (one RMW, two MW) expressed financial restrictions related to buying soap.

The main sources of water used for bathing were tube wells, ponds, rivers, and occasionally toilets. A common practice among participants was a ritual bath at the start of menstruation, during which they wash their body and hair. Women needed others to retrieve water for them; they were not allowed to touch water given perceptions that doing so during this state would contaminate the water source. If menstruation began at night, women would bathe immediately, provided support or water was available and it was not too late, or would wait until the morning, often sequestering themselves in a corner of a room to limit their polluting state from affecting anything or anyone around them. Accessing water late at night was a challenge because women had to navigate difficult terrain in the dark and awaken others to help them. One UMW reported preemptively storing water in anticipation of menstruation starting at night. Seasons further complicated bathing practices. In the winter, bathing at nighttime was very cold. During monsoons, women got wet while finding a location to bathe and felt dirty, even after bathing, due to the water and mud outside. In the summers, ponds dried up and left women with fewer places to bathe.

**“Having access to facilities to dispose of used menstrual management materials.”** Women in this study did not report accessing specific facilities for disposal. Rather, they disposed of their menstrual materials in a variety of places outside of the home and in the surrounding environment, including the pond, river, jungle, or buried in the ground. MW shared that they disposed of cloth after two to three months of use and buried it in the swampy area of the pond. A few women disposed of their materials in the toilet:

*So*, *I dispose them [pads] in the latrine…(laughs)*, *where would I drop them otherwise*? *I drop them in the latrine and put water*. *(FLI*, *RMW*, *Toilet)*

**“They understand the basic facts linked to the menstrual cycle and how to manage it with dignity and without discomfort or fear.”** Women in this study had many questions about the menstrual cycle. They sought clarity about pain and menstrual irregularity and worried that something might be wrong with their bodies. Women did not have information as to why their period might be late, at times even asking the Research Assistant for information:

*If it does not happen on date then is it a problem*? *(FLI*, *UMW*, *Toilet)*

### Concerns extending beyond JMP definition

**Fig 1:**
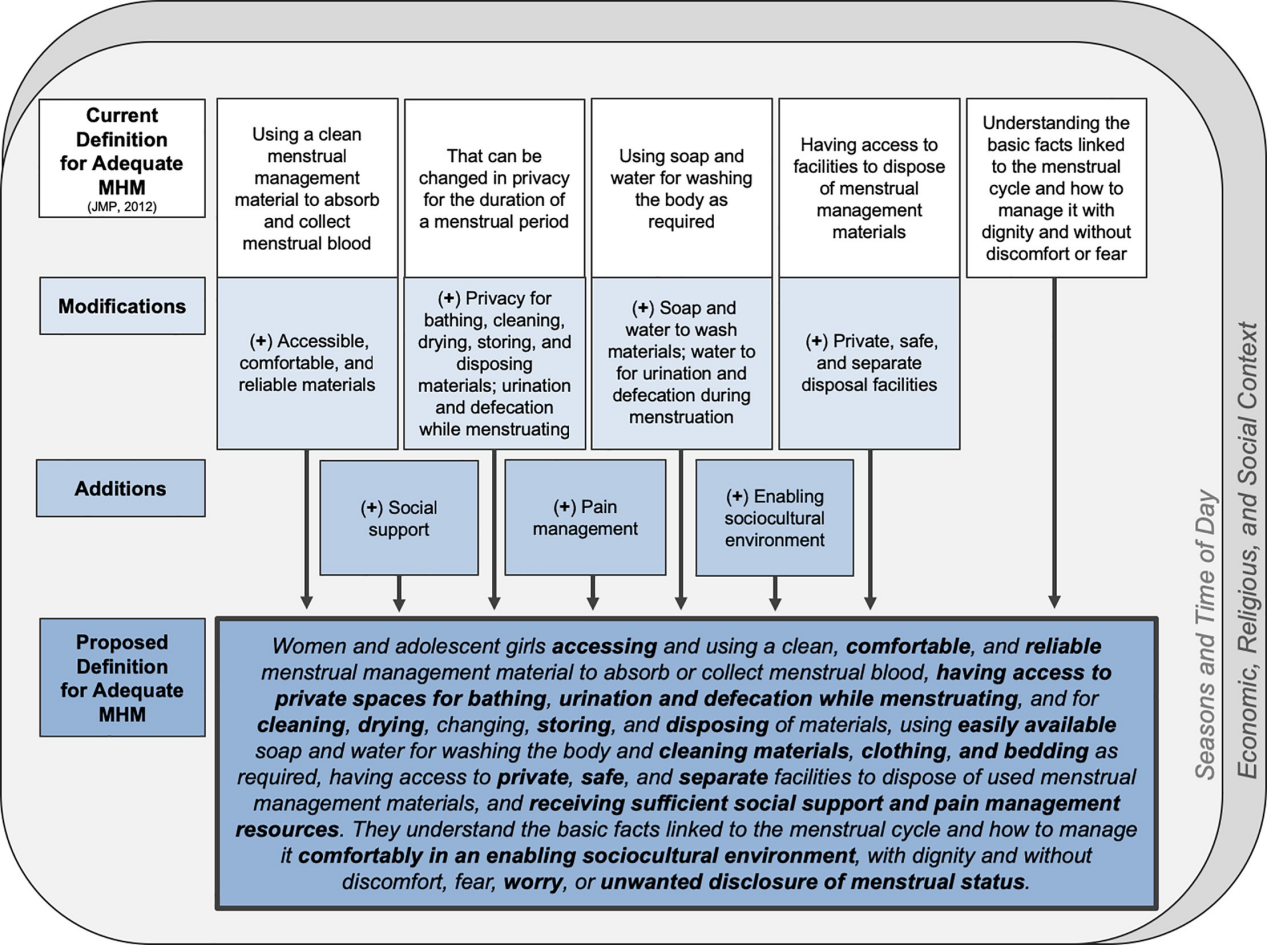
Conceptual framework of proposed definition for adequate menstrual hygiene management for women in Odisha, India.

In addition to the needs for MHM reported above, practices and concerns emerged from the data that extend beyond the requirements codified in the JMP definition. These additional concerns are discussed below and visualized in Fig 1 as both modifications to portions of the existing definition and further additions to it.

**Modifications to existing JMP definition for adequate menstrual hygiene management. Needs and concerns for materials beyond cleanliness and absorbency:** Women had significant concerns beyond the cleanliness of menstrual materials. While the JMP definition indicates that materials should collect and absorb blood, there is no consideration for how reliable, comfortable, or compatible these materials should be with women’s lives, or even if they be accessible. Women worried cloth would change positions, fall out of their underwear, or become saturated while moving or sleeping, causing stains on clothes and bedding. Women expressed concern and embarrassment about others seeing any menstrual leaks. To address this, some tied a string to the cloth and then tied the string tightly around their waists to keep the cloth from falling. UMW reported repeatedly looking at their backs to see if there were any stains.

Materials impacted women’s management of other needs, frustrating the processes of urinating and defecating. Women had to remove cloth from their underwear and either hold it in their hands or risk it falling out of their underwear:

*If we would be urinating and wear a cloth*, *if someone comes and we suddenly get up*, *as it would be placed on the panty if we get up it might fall*, *so someone gets to see*. *(FGD*, *UMW)*.

The use of pads depended on availability and accessibility, as many women could not purchase pads, due to markets being as far as 5–6 kilometers away, or had household level economic restrictions:

*We are poor people*. *So father cannot provide pad*. *How can we say that we have to bring pad with father’s money*? *We can’t say*. *We bring occasionally during rainy season*, *other times we adjust*. *(FLI*, *UMW*, *Toilet)*

RMW faced specific barriers to pad access. Due to mobility restrictions, one RMW depended on others to purchase pads and because she felt embarrassed to request them, she used cloth available at the house. Another RMW had to switch to using cloths after marriage because she moved to a new village that did not sell pads.

**Privacy needs and concerns for behaviors beyond changing:** Beyond the need for privacy to change materials, women articulated the desire for privacy for bathing, washing, drying, storing, and disposing of materials, urination, and defecation.

Women were cognizant of the best times to bathe at the pond when other people, particularly men and boys, were not around. Women were careful to wait until people, specifically males, were not in the area prior to washing. This waiting was an additional challenge for women with children, as they had to leave home for an extended period of time:

*That males do not get to see*, *that is our method*, *and in doing that method there is inconvenience*, *sometimes we are seen*, *that is the thing…No there is no isolated place*. *Whatever we will do*, *we will do in the open*. *(FGD*, *MW)*

Women expressed concern about bathing in public twice in one day, as that would indicate they had started menstruating. To hide their menstrual status, a few women would justify the additional bathing by telling others they had stepped in dog feces. Despite the challenges of accessing water at night, some women preferred to bathe in the cover of darkness to enable privacy.

When washing their cloths, some women poured water on the ground around the tube wells to conceal any lingering signs of blood. If other people were using the tube well, some MW reported hiding their used cloth in dirty places to wash at a later time noting they could not keep it in the house:

*Participant 3*: *Will take it out and keep it separately*, *cannot keep it inside the house*.

*Participant 4*: *It has to be kept at a separate place*, *be it amidst cow dung heaps or in the firewood accumulated for lighting stove*. *Is that easy*? *(FGD*, *MW)*

While washing her cloth at the tube well, one UMW hid her cloth behind a bucket, behind her back, or under her foot if her father or brother walked by; one MW would avoid using the tube well to wash her cloth altogether because her father-in-law was typically nearby. Some women washed their cloth in the toilet or bathroom; one RMW would dispose of cleaning water in the toilet.

A predominate challenge for women across life stages was drying cloth after washing. Women wanted to hide their cloth from men to prevent shame and, in doing so, felt they may be using dirty, germ-infested locations:

*There is no place to wash and dry it*. *That when I would be hanging it to dry*, *elder and younger brothers-in-law*, *father-in-law may be around*. *Will I not feel shy*? *Where will I take it to a secluded place and dry it[*?*] So we face a lot of problem*. *(FLI*, *MW*, *Toilet)*

*Participant 5*: *…When we hang it to dry outside*, *the dust falls on it and whether someone will see and we will feel ashamed so we have to dry it elsewhere*.

*Participant 4*: *Just because people will get to see*, *we dry it in a dirty place*.

*Participant 5*: *Are there germs in it or not…Hide and dry*. *(FGD*, *MW)*.

Women hid drying cloths on the roof, in bamboo bushes, in the cow shed, in dark places inside their homes, and underneath other clothing. Women understood that drying cloth in the sunlight could prevent germs from developing on the cloth that may result in infection, yet the need to hide cloths prevented this practice. Additionally, women had concerns about ants, scorpions, and other creatures getting in the cloth while it was drying and then entering their bodies.

Seasons further complicated private drying practices. Women found drying cloth during the summers to be quick, allowing for more regular washing, but were concerned about drying materials during monsoons. Cloth would not dry if outside in the rain, protected places to dry cloth were limited, and cloth could become dirty again due to weather conditions:

*But is it not difficult for drying*? *Does it dry instantly in monsoon*? *When the wind blows*, *it may fly away and fall somewhere*. *Is it not a concern*? *While in summer you can dry it and fold and keep*. *(FLI*, *MW*, *Toilet)*

Sometimes cloth would not dry out at all and since wearing wet materials felt dirty and uncomfortable, some women would use pads specifically during monsoons. Between periods, some women hid menstrual materials by wrapping their cloth in plastic and placing it in the straw roof.

Beyond the absence of specific disposal facilities, women perceived harms connected to their lack of privacy for disposal and took specific steps to mitigate those harms. One MW worried her disposal practices would identify her because she was the only one using pads in her household. Materials were disposed of in places where no one would see them, such as wrapped in paper in the jungle or in the muddy part of the pond:

*The big pond that we have…We throw it there*. *There is too much sludge…If we take a stick and press it there*, *it will go that side*. *(FLI*, *RMW*, *No toilet)*

Although disposing pads in bodies of water was a common practice, women felt dirty seeing them resting on the pond or river banks:

*In our village all those who are using sanitary napkins are throwing it in the river*. *They are not burying it anywhere*, *there is no particular place to bury*. *(FGD*, *UMW)*

Women feared germs from the pads would enter the water and cause infections. A few women (UMW, MW) were told not to throw pads outside because if animals smelled it, increased bleeding or infection may result. Another MW believed if a snake touched her cloth, she would be unable to have a child. This fear of animals accessing menstrual materials was shared; an UMW threw materials in the pond so dogs could not access them and expose her:

*Even for disposing we need a space*, *there would be people and if dogs will drag it somewhere we will have inconvenience*, *so have to worry*, *have to look for a place to dispose*. *(FGD*, *UMW)*

Women desired private locations to urinate and defecate while menstruating because they needed to remove their cloth and may have visible signs of blood on their hands and legs. Women brought extra water to urinate and defecate during menstruation in order to rinse away any blood that remained on the ground. A few MW demonstrated how they would rub the ground with their feet to obscure the blood. Women worried others would see the blood or become infected by touching it.

**Soap and water needs and concerns beyond bathing:** The JMP specifically articulates the need for soap and water for washing the body during menstruation, however we found the need for soap and water extends to washing clothing, bedding, and materials, and managing urination and defecation while menstruating, as noted above. For those who used a tube well to wash their cloth, some felt it was dirty to wash near where people fetched drinking water, so they would carry water to a different location. The distance to the pond was a concern for washing cloth, given the three to four times some women traveled there each day. One UMW preferred the flowing water of rivers because she could clean herself, her cloth, and urinate at the same time. Despite the other challenges monsoon season brings, one RMW found washing cloth was easier in the monsoons because the ponds were full of water.

Women often felt dirty from washing their cloth. One UMW felt she polluted the pond and was concerned for others who used the same water. Others did not like touching the cloth with their hands and felt dirty even after washing with soap. In the absence of soap, one MW used dirt to clean her cloth.

In addition to washing the clothes they were wearing at menstrual onset or that may have been stained, women discussed the need to wash all their sheets and blankets if menstruation started while they were in bed, regardless of whether or not they were actually soiled. This washing extends women’s needs for water and soap. Some women slept outside of their beds if they anticipated their period may be coming to prevent the work of washing bedclothes.

**Additions to JMP definition for adequate menstrual hygiene management.** In addition to our proposed modifications to the existing JMP definition, we identified notable needs and concerns not yet captured. Concerns surrounding physical pain and the need for social support were expressed by women in this study and are presented below as additions to the definition for adequate MHM. Further, we note how an unsupportive sociocultural environment is an underlying driver of many of the concerns and challenges that women have noted and requires attention.

**Discomfort and pain:** The JMP definition does not address pain and discomfort during menstruation, however a general sense of discomfort was frequently expressed by women in this study. Women across life stages gave accounts of bodily pain during menstruation including stomach aches, headaches, vomiting, and pain in the legs, hands, and back. Some women expressed how this pain negatively affected their ability to complete regular household tasks (e.g., cook, harvest crops), maintain healthy behaviors such as eating and sleeping, move normally (e.g., ride a bike), and care for their children, which could further result in “tension” or stress:

*No*, *if food and sleep are a problem*, *then it is a tension*. *And if you can’t work then that is a tension*. *My waist is paining and I can’t go to work*. *Then what will my kids eat*, *how will they live*? *That is a tension*. *(FLI*, *OW*, *Toilet)*

UMW worried their expressions of pain would disclose their menstruation status to other family members:

*When you have these stomach cramps everyone will come to know*. *All in the family will know*, *isn’t that embarrassing*? *That is a big concern*. *(FGD*, *UMW)*

For some, the physical and emotional burdens of menstruation led to isolation:

*Participant 2*: *In normal times we can roam about freely*, *we can go wherever we want*. *But when we have our periods we don’t feel like going anywhere*.

*Participant 1*: *Do not feel like going anywhere…Awkward*.

*Participant 5*: *Meaning there is a different feeling*.

*Participant 3*: *It’s like a burden on the head*. *(FGD*, *UMW)*

While the experiences of women in school were not widely explored in this research, some of the unmarried women explained how pain challenges their academic requirements:

*I am not able to attend my classes*. *I don’t feel comfortable so I don’t go for my classes*, *in fact I avoid going out anywhere*. *(FLI*, *UMW*, *Toilet)*

The articulation of clean and absorbent materials in the JMP definition also does not account for the ways in which materials can cause pain or impede women’s ability to move and carry out work. Women experienced discomfort using a cloth–it smelled and felt thick and heavy in their underwear–making sitting and standing difficult. The edges of cloth rubbed the sides of women’s legs causing swelling, rashes, and wounds:

*In these summers*, *I get scratches as edges of dry cloth rubs with my body*. *I feel why is this badi poda [cursed thing] happening always*? *I am getting scratches*. *And the thread that we wear on the waist to tie the cloth*, *becomes very tight and I get scars and become red in colour*. *(FLI*, *MW*, *No toilet)*

This pain was felt more acutely during the summers, when heat and sweat exacerbated the rubbing and sensitivity. Some mentioned pain restricted mobility and productivity, as the rubbing increased with movement:

*It will hurt*. *The edges of the dry cloth will rub with my body and then I would be sweating*. *I will have to do all household work and it would be hurting*. *(FLI*, *OW*, *No toilet)*.

**The need for social support:** Women of all life stages required the support and physical assistance of others during menstruation, which is not accounted for in the JMP definition:

*Participant 1*: *We are at the mercy of others*.

*Participant 6*: *We have to depend on others*, *is there an option*? *(FGD*, *MW)*

Women relied on mothers, mothers-in-law, sisters, sisters-in-law, and children to retrieve water from the tube wells, as they were restricted from touching the pump while menstruating. RMW had additional mobility restrictions that contributed to their need for others during menstruation:

*For water* …*Being a recently married woman I can’t go out*. *I have to depend on someone to fetch water and provide me so that I can wash my clothes or clean myself or bathe*. *So we are dependent on others*. *(FLI*, *RMW*, *Toilet)*

At the onset of menstruation, women did not touch their clothing until after bathing, so another person carried their clothes for them. At night, women needed accompaniment to the pond or tube well to bathe. Women shared fears about ghosts, dogs, snakes, darkness, and other people at night, and asking others to join mitigated this fear. Women, particularly UMW and RMW, felt badly if they woke sleeping family members for assistance. At times, family members were resistant to help, so women waited outside until morning to bathe. Some women reported feeling shy asking for this help, as it alerted others to their menstruation status and thus impeded their privacy.

Reliance on others extended beyond washing and bathing. Women were restricted from entering the kitchen, preparing food, and touching utensils while menstruating, so others in the household completed this work. Additionally, women were restricted from participating in puja, daily rituals in the home performed by women, during menstruation and needed others to perform it on their behalf:

*During that time you have to go around pleading people…To perform the Puja at home…Early in the morning I have to go and request someone*, *may be a girl in our neighbourhood to perform the puja* … *(FLI*, *UMW*, *Toilet*)

**The need for an enabling sociocultural environment**: The JMP definition of MHM does not directly address the sociocultural environment within which women manage menses, yet this permeates the range of challenges and concerns voiced by women. Women’s inability to manage menstruation is constrained by their inability to control resources (e.g., to purchase materials and soap), move freely (e.g., to access markets, get water, find privacy to bathe and wash, socialize), and make decisions about and independently act on their personal needs (e.g., to bathe without need for social support). Further, the sociocultural perception of menstruation as polluting further drives women’s behaviors and compromises their ability to manage. Women wait for privacy or seek alternate locations to wash materials in private, dry cloths in less optimal areas so they and their materials are unseen, hide materials in dirty locations, and carry extra water to erase signs of blood, all in an attempt to keep their polluted menstrual status hidden. This polluted status drives women to do more work washing bed clothes that are unsoiled, sleep outside of their beds at night, and recuse themselves from social interactions, including schooling. This status also imposes rules and restrictions upon them, requiring them to bathe at menstrual onset, limiting their ability to touch water, touch food or cook, or perform religious acts or enter temples. This sociocultural environment, therefore, can act as a barrier to management even if resources and facilities are available.

## Discussion

We applied the definition of MHM to determine if it adequately and comprehensively applied to women’s lived experiences in rural Odisha, India. The findings from this research demonstrate that women in rural Odisha face the challenges to adequate menstrual hygiene management codified in the JMP definition [47]. Further, these women expressed needs that extend beyond those articulated in the definition, suggesting that a modified definition for MHM among this population is warranted. We acknowledge the logistical challenges of suggesting a modified definition for MHM, including potential measurement complications of adding more details to be assessed. However, definitions designed to inform research require testing in order to determine if they are adequate. If not, untested definitions may motivate research trajectories that fail to adequately assess lived experience. Specific quantitative measures of MHM per this definition have yet to be codified and used widely, making an evaluation of the definition all the more timely in both our population of interest and elsewhere.

Below and visually depicted in Fig 1, we propose an amended definition for adequate MHM for women in this population that captures the breadth of women’s needs uncovered in this study. Several of these needs and concerns have been noted elsewhere, suggesting applicability to other populations and contexts:

*Women and adolescent girls* *accessing* *and using a clean*, *comfortable*, *and* *reliable* *menstrual management material to absorb or collect menstrual blood*, *having access to private spaces for bathing*, *urination and defecation while menstruating*, *and for* *cleaning*, *drying*, *changing*, *storing*, *and* *disposing* *of materials*, *using* *easily available* *soap and water for washing the body and* *cleaning materials*, *clothing*, *and* *bedding* *as required*, *having access to* *private*, *safe*, *and* *separate* *facilities to dispose of used menstrual management materials*, *and* *receiving sufficient social support and pain management resources*. *They understand the basic facts linked to the menstrual cycle and how to manage it* *comfortably in an enabling sociocultural environment*, *with dignity and without discomfort*, *fear*, *worry*, *or* *unwanted disclosure of menstrual status*.

Beyond clean materials, women noted access constraints and articulated the need for materials that are comfortable, reliable, and compatible with their responsibilities and movements. Unmarried and recently married women in this population face social and financial restrictions to acquiring pads, requiring them to seek support to get materials they want. Such barriers have been noted in other research in India, Tanzania, among others [8, 31, 45]. Research from Kenya found women and girls engage in transactional sex to access pads because of economic barriers, putting them at risk for sexually transmitted infections [9, 56]. While the Indian and Kenyan contexts are quite different, both scenarios show how women and girls lack agency to independently act to get resources that serve their life needs.

Even if women have access to materials to manage menstruation, what they have may not be good quality. Women in Odisha were specifically concerned about the reliability of materials to prevent leaks and stains, the comfort or lack thereof that they may experience, and compatibility of their materials with other daily needs, including mobility, work, urination, and defecation. Concerns about the reliability of materials, whether cloth or commercial products, have been discussed widely in research with girls in school who describe distraction and reduced participation because they are worried about leaks that could reveal their menstrual status and cause shame [4, 21, 57]. Similar to findings from this study, research with adolescent girls in Kenya has also documented how materials can be painful, itchy, and cause rashes and sores, representing an unnecessary additional burden to have to cope with regularly for a normal part of life [57].

The need for privacy beyond changing and inclusive of bathing, urination and defecation while menstruating, and washing, drying, and storing cloth, has been noted by other studies in India and Odisha specifically [8, 44, 45]. Women in this study attempted to hide their menstrual status from men, particularly when bathing in the pond, however additional restrictions may exist for women as they seek to hide menstrual status from boys as well. The lack of privacy for these management activities and for coping with pain threatens women’s ability to keep their menstrual status hidden, resulting in feelings of shame. As Sommer et al (2015) have noted, this shame is common across contexts and has been “perceived as an inevitable part of the social order”, and thus has garnered less attention and action [11]. However, as Sclar et al (2018) find from their meta-synthesis, a lack of sanitation privacy can influence women’s well-being, so it follows that a lack of ability to conceal menstruation due to pain, washing, leaks, and restricted behaviors is likely to do the same [58]. In our population, women did not use sanitation facilities during menstruation for urination, bathing/washing, of for cleaning or changing materials, despite the potential privacy these facilities could afford—a finding that diverges from research among women’s use of sanitation facilities in Nigeria [59]. The social norms around latrine use in rural India, including the perception that these facilities are dirty, is likely more powerful of a barrier to use than the potential draw to find privacy within [60]. Bathing areas that are separate from latrines have been associated with well-being among this population and may better provide the infrastructure for private management activities [48].

Moreover, the coping practices that women adapted to keep their menstrual status hidden from family members, men, and boys constrained their abilities to adequately manage, resulting in drying cloth in dark and dirty places, disposing cloth in hidden places outside where no one could see or come in contact with them, and urinating and defecating with additional concerns of menstrual blood being seen. These coping practices could be harmful. Women in this study worried about infection resulting from drying cloth in hidden and dark places, which is consistent with findings reported from India, Tanzania, and South Asia [6, 8, 12, 20]. Studies have explored the connection between the type and condition of materials with urogenital infections, and while this relationship has not been substantiated, the effects of this concern were felt among participants and warrant further exploration [6, 27–29]. And, these coping practices take work. As reported in another study in India, hiding signs of menstruation from men made washing cloth more difficult [61]. Further, the search for privacy takes time from women due to increased waiting times at water sources for bathing, washing, and getting water to manage urination and defecation while menstruating so blood can be washed away.

Our findings contribute detailed accounts of disposal practices and further demonstrate the need for access to “female-friendly toilets” with disposal bins inside [8, 14, 21, 62]. No women in our study reported disposing of menstrual materials in specific facilities. Instead, they would dispose of old cloths and pads in bodies of water, the jungle, the mud, or the latrine in order to maintain privacy, consistent with other studies of rural women in India [8, 63, 64]. Women were concerned about this practice due to perceived harm they felt these materials could do if others came into contact with them. Environmental impact and threats to latrines and sanitation systems are already a concern, however as more women transition to commercial and non-biodegradable products for managing menstruation, latrine fill up and environmental accumulation will be increasingly urgent [63, 65, 66].

A focus on private, safe, and separate disposal facilities, where materials can be confined and out of view of others, may reduce women’s concerns about menstrual materials being accessed by animals, contaminating others through contact, or revealing their menstrual status if seen in the open. Concerns for appropriate disposal have been voiced by girls in Ghana who worried about infertility if animals touched their pads and girls in Bolivia who believed they could get cancer if they burned their menstrual blood [12, 67]. Further research is needed to identify solutions for disposal that account for women’s needs, the cultural context, and the environment.

Women provided detailed accounts of physical pain during menstruation, including both menstrual pain and the previously noted pain from uncomfortable materials, which affected women’s ability to move, work, and eat normally. Importantly, women noted how their lack of ability to manage pain could make their menstrual status known, leading to shame. Menstrual and material-induced pain have yet to be widely explored in low and middle income contexts specifically, apart from a few studies that highlight menstrual pain among a range of concerns for girls [21, 61]. Research from other fields has found women’s reports of pain to not be taken seriously, causing them to endure pain unnecessarily for extended periods or to not receive needed treatment [68]. It is important that this sector not contribute to the silencing of women’s pain and including it in a revised definition is one way of signaling that these concerns are being heard and are worthy of addressing.

Women in this study experienced a range of restrictions during menstruation and relied on others to manage their household and menstruation-related obligations. Religious restrictions, physical separation, and inability to touch food during menstruation were consistent with findings among other women in India and schoolgirls in Kenya [8, 26, 44]. Restrictions related to menstruation permeate other facets of life and can result in missed opportunities. Notably, research in Bangladesh found girls who had restrictions imposed on them were at greater odds of missing school during their periods [3].

Many researchers have explored girls’ fear and confusion at menarche due to insufficient information and preparation [4, 12, 21, 23, 67, 69]. However, our findings present another dimension of inadequate information about menstruation, specifically regarding irregularity and menstrual pain, both of which led to fear and worry for women in this study that something was wrong with their bodies. Such concerns may also be attributed to perceptions of regular menstruation as symbolic of women’s abilities to be fecund and therefore their ability to be good wives and mothers [34]. Knowledge about the process of menstruation that includes information beyond the most typical patterns and how to manage menstrual pain could help improve women’s experience of MHM.

### Strengths and limitations

A key strength of this study is its reliance on two methods of qualitative data collection: individual interviews and FGDs. The FGDs took place following the FLIs and corroborated findings from the interviews, contributing to the study’s validity. This study engaged rural women in Odisha exclusively and the findings are limited to this population, however the diversity captured in the sample provides a broad range of experiences of women at different ages, marital statuses, latrine and water access, and castes that allowed for variation in the data. Moreover, this research gives voice to the experiences of rural women who are underrepresented in studies on menstruation. In an analysis of data across eight countries (including India) from the Performance Monitoring and Accountability 2020 survey programme, Hennegan et al (2018) found that women who were wealthy, urban, educated, young, and unmarried were more likely to be eligible to answer questions about menstruation, resulting in an underrepresentation of data from rural, older, less educated, and married women [70].

While various castes and tribes were represented in the study population, we did not intentionally sample to have castes equally represented or include specific questions about caste to enable identification of varied practices, experiences, and concerns by caste. Future research could seek to identify how caste may influence women’s menstruation experiences. RMW did not participate in FGDs, however they are represented in the FLI sampling frame (12 interviews with RMW), so we do not believe this exclusion impacted our findings. The FGDs had mixed-caste groups, which may have affected the openness to which participants expressed their thoughts. For each life stage, not all caste categories were represented, which may limit the breadth of the results. Finally, this research did not capture the experiences of adolescent girls, so it could not compare their experiences to those of the women in this study. This comparison could yield new findings and is recommended as an area of further study.

## Conclusion

Outside of a few key papers that highlight the menstrual experiences of women in the workplace, emergency context, or vaginal bleeding throughout the life course, experiences of menstruation among adult women and outside of school settings remains underexplored [38, 39, 43, 70]. This research confirmed that women need tangible support to manage menstruation, however we uncovered needs for MHM that extend beyond those identified in the JMP definition. Concerns connected to psychosocial support, agency, gendered environments, the role of men and boys, and social norms persist even if menstrual management needs are met [44, 45, 71]. Experiences of shame, restrictions, and fear may be hard to capture in a definition, however are integral to women’s experience of menstruation as well as their overall health and well-being. Our expanded definition for adequate MHM can inform monitoring efforts, program priorities, measures of assessment, and future research to account for the scope of menstrual management needs of women in Odisha, and potentially beyond. Researchers have previously engaged the JMP definition quantitatively to guide research and assessment [72], however our definition, though particularly suitable to the Indian context, may be more comprehensive in other locations as well since many of our modifications and additions have been identified in studies from various locations.

We recommend additional research in different contexts to discern the wider validity and applicability of this definition. Given our findings, we posit that a broader definition for MHM may better capture the range of menstruation-related needs of women and further the discourse on MHM in the sector and is an important first step for creating appropriate measures to assess the extent that these experiences and circumstances may actually contribute to negative health outcomes.
